# Synergistic Modulation of Cellular Contractility by Mixed Extracellular Matrices

**DOI:** 10.1155/2012/471591

**Published:** 2012-11-29

**Authors:** Aastha Kapoor, Shamik Sen

**Affiliations:** WRCBB, Department of Biosciences & Bioengineering, IIT Bombay, Mumbai 400 076, India

## Abstract

The extracellular matrix (ECM) is known to provide various physicochemical cues in directing cell behavior including composition, topography, and dimensionality. Physical remodeling of the ECM has been documented in a variety of cancers. In breast cancer, the increased deposition of matrix proteins, their crosslinking, and alignment create a stiffer microenvironment that activates cell contractility and promotes cancer invasion. In this paper, we sought to study the collective influence of ECM composition and density on the contractile mechanics of human MDA-MB-231 cells making use of the recently established trypsin deadhesion assay. Using collagen and fibronectin-coated surfaces of varying density, we show that cell contractility is tuned in a density-dependent manner, with faster deadhesion on fibronectin-coated surfaces compared to collagen-coated surfaces under identical coating densities. The deadhesion responses are significantly delayed when cells are treated with the myosin inhibitor blebbistatin. By combining collagen and fibronectin at two different densities, we show that mixed ligand surfaces synergistically modulate cell contractility. Finally, we show that on fibroblast-derived 3D matrices that closely mimic *in vivo* matrices, cells are strongly polarized and exhibit faster deadhesion compared to the mixed ligand surfaces. Together, our results demonstrate that ECM composition, density, and 3D organization collectively regulate cell contractility.

## 1. Introduction

Of the several hallmarks of tumor formation, the extracellular matrix (ECM) plays a central role in regulating evasion of apoptosis, uncontrolled proliferation, angiogenesis, and metastasis [1, 2]. The acquisition of these hallmarks is made possible through a series of continuous alterations in ECM composition and organization during tumor progression that is manifested in altered ECM mechanical properties. For example, tumors are significantly stiffer than normal tissue, and malignant transformation may be promoted by ECM stiffening. Such alterations in ECM properties lead to altered tensional homeostasis, that is, the force balance between individual cells and the ECM [3].

The ECM is composed of a heterogeneous network of collagen, fibronectin, laminin, glycoproteins, and proteoglycans, with its composition varying in a tissue-specific manner. ECM composition and organization are frequently altered in the context of cancer. For example, increased deposition of collagen I is associated with mammographic density and an increase in the development of breast cancer [4]. Further, lysyl oxidase-induced crosslinking of collagen leads to stiffening of the tumor stroma and induces tumor progression [5]. In addition to increased deposition and crosslinking of matrix proteins, collagen fibers undergo reorganization from a random network to tracks of aligned fibers that promote cancer invasiveness [6, 7]. Such alterations in ECM density and alignment have been documented in a wide variety of epithelial cancers including breast cancer, prostate cancer, and ovarian carcinomas [8]. Stromal fibroblasts in the tumor microenvironment are also known to secrete an aligned matrix rich in fibronectin and collagen. Moreover, fibronectin deposition has been implicated as an early step in metastasis [9], and fibronectin is known to modulate collagen fibril organization by directly binding collagen [10]. Collectively, these studies indicate that increased density and alignment of collagen and fibronectin in the ECM lead to increase in ECM stiffness which drives tumor progression. 

Numerous biophysical studies have focused on understanding how ECM features, namely, ECM stiffness and ECM density, influence cellular processes including cell spreading and motility, both in normal cells and in cancer cells. Spreading and motility of 3T3 fibroblasts were demonstrated to exhibit biphasic dependence on collagen I density, with the threshold density comparable to the surface density of integrins expressed by these cells [11]. Similar biphasic dependence of cell spreading and motility has been observed in smooth muscle cells cultured on ECM-coated stiffness-modulated polyacrylamide hydrogels, where the optimal ECM stiffness for spreading was seen to depend on ECM density [12, 13]. In contrast to the biphasic spreading response observed in fibroblasts and smooth muscle cells, bovine aortic endothelial cells (BAECs) were seen to spread increasingly with increase in ligand density on RGD-functionalized polyacrylamide hydrogels. Moreover, the mode of cell spreading was found to change from anisotropic spreading on low-density surfaces to isotropic spreading on higher-density surfaces [14]. Similar ECM density-dependent spreading response has been reported in breast, lung, and prostate cancer cells [15]. In addition to illustrating the coupled dependence of cell spreading on ECM stiffness and ECM density, these results highlight cell type-dependent differences in cell sensitivity to changes in ECM stiffness and/or ECM density.

Concomitant with ECM-dependent cell shape changes, alterations in the ECM mechanical properties are also closely tied with alterations in cancer cell mechanical properties. Increased traction forces have been reported in metastatic breast, lung, and prostate cancer cells compared to noninvasive cells with increase in ECM density [15]. Measurement of 3D traction forces in collagen gels revealed a hypercontractile phenotype of breast and lung cancer cells compared to their nontransformed counterparts [16]. Furthermore, breast cancer cells possessing ErbB2 transforming potential embedded in 3D matrices were found to stiffen in response to elevated matrix stiffness [17]. In contrast to the above results which are reflective of increased stiffness of cancer cells, bladder cancer cells are significantly softer than normal cells [18]. Cell softening has also been reported in pancreatic cancer cells in the presence of the bioactive lipid sphingosylphosphorylcholine (SPC) that is known to play an important role in pancreatic cancer metastasis [19]. Together, these results illustrate that change in the mechanical properties of cancer cells may involve either cell stiffening or cell softening, possibly dictated by the biomechanical properties of the microenvironment.

The close correlation between cancer cell mechanical properties and cancer invasiveness has fueled tremendous interest in developing techniques for measuring the mechanical properties of cancer cells. In comparison to some of the widely used techniques used for measuring cell mechanical properties which include atomic force microscopy (AFM) [20–23], optical traps [24], magnetic twisting cytometry [25–27], and micropipette aspiration [28, 29], trypsin deadhesion assay is one recently developed rapid and inexpensive technique that can be used for probing the contractile mechanics of adherent cells [30]. In this assay, the cellular tensional homeostasis is gauged by tracking the kinetics of cell detachment from their underlying ECM upon treatment with the enzyme trypsin. The kinetics of cell detachment exhibits sigmoidal behavior with two time constants that scale inversely with cortical stiffness values obtained using the AFM. Using this assay, human brain tumor glioma cells cultured on fibronectin-coated substrates were found to exhibit ECM density-dependent deadhesion dynamics with the fastest deadhesion observed on the highest-density surfaces [31]. Interestingly, deadhesion time scales were found to be more sensitive to changes in contractility compared to AFM stiffness measurements illustrating the utility of the deadhesion assay in quantifying alterations in cancer cell mechanics driven by cytoskeletal remodeling.

In this paper, we have studied the collective influence of ECM composition and ECM density on the contractile phenotype of cancer cells making use of the trypsin deadhesion assay. Using the highly metastatic MDA-MB-231 human breast cancer cell line, we have measured the spreading and deadhesion dynamics on engineered ECMs of varying composition and density, as well as on cell-derived *in vivo* mimetic ECMs. First, while cell spreading on glass coverslips coated with varying concentrations of collagen or fibronectin was found to depend weakly on ECM density, cell shape was found to depend both on ECM density and on ECM composition. In comparison to cell spreading, deadhesion time scales exhibited a much stronger dependence on ECM density with faster deadhesion observed with increasing ligand concentration on both collagen- and fibronectin-coated substrates. Similar results were also obtained on hybrid matrices fabricated by combining collagen and fibronectin, where increase in ECM density led to faster deadhesion. Compared to hybrid matrices, even faster deadhesion was observed on NIH 3T3-derived *in vivo* mimetic 3D matrices. Taken together, our results suggest that the mechanical properties of breast cancer cells are intimately tied to ECM composition, density, and organization and illustrate the utility of the trypsin deadhesion assay in measuring ECM-dependent alterations in the mechanics of cancer cells. 

## 2. Materials and Methods

### 2.1. Preparation of ECM-Coated Glass Coverslips

Glass coverslips were ethanol-sterilized and incubated with rat tail collagen I and/or human plasma fibronectin (from Sigma) at theoretical densities of 0.01, 0.1, 1 and 10 *μ*gm/cm^2^, respectively. After overnight incubation at 4°C, samples were blocked with 1% Pluronic F127 (Dow) for 10 minutes and rinsed with PBS before plating cells. 

### 2.2. Measurement of ECM Coating

The surface adsorption of ECM proteins at different densities was assessed by comparing the absorbance of the stock solutions with that of scraped solutions obtained by scraping the contents of the coverslips and reconstituting in equal volume of buffer as that of the stock solutions. For this, the Bradford reagent (Sigma) was combined with the solutions in a 1 : 1 ratio and 20% SDS added to the solutions in 1 : 100 ratio to increase the absorbance readings [32]. Absorbance readings were obtained for both stock and scraped solutions using an UV spectrophotometer (Jasko) at 595 nm. The experiment was repeated thrice for all the conditions. Student's *t*-test was used to determine statistical significance between absorbance values across different coating densities.

### 2.3. Preparation of Fibroblast-Derived Matrix

NIH 3T3 cells were cultured in high-glucose DMEM containing 10% fetal bovine serum (FBS), 100 units/mL penicillin, and 100 *μ*g/mL streptomycin for more than 20 passages prior to matrix production. Cell-derived matrix was obtained by treating confluent NIH 3T3 cultures with fresh maintenance media supplemented with 50 *μ*g/mL cell culture-tested ascorbic acid (Sigma) every other day for 8 days [33, 34]. After eight days of ascorbic acid treatment, cell-derived matrix was obtained by removing the fibroblasts through alkaline detergent treatment (0.5% (v/v) Triton-X-100 and 20 mM NH_4_OH) and gentle washing with PBS. MDA-MB-231 cells were grown on fibroblast-derived matrices for 24 hours prior to spreading and deadhesion experiments.

### 2.4. Cell Culture

MDA-MB-231 cells were obtained from NCCS (National Centre for Cell Science, Pune, India) and maintained as monolayer culture in a humidified atmosphere of 95% air and 5% CO_2_ in L-15 media (Leibovitz15, HiMedia) supplemented with 10% fetal calf serum (HiMedia) and 1% penicillin/streptomycin antibiotics (PenStrep, HiMedia). Cells were maintained in 25 cm^2^ cell culture flasks (Corning), harvested with 0.25% trypsin-EDTA (HiMedia), and passaged every 3-4 days. For experiments, cells were plated on glass coverslips coated with ECM at varying densities, and spreading and deadhesion were assessed at a 24-hour time point. 

### 2.5. Immunofluorescence Labeling

For staining, cells cultured on different substrates were washed with PBS, pH 7.4 (HiMedia), permeabilized using permeabilization buffer (10 mM HEPES, pH 6.9, 50 mM NaCl, 3 mM MgCl_2_, 0.5% Triton X-100, 300 mM sucrose, 1 mM EGTA, and protease inhibitor cocktail (Catalog no. P8340, Sigma)) to flush out cytoplasmic proteins, and fixed with 4% paraformaldehyde (Sigma) in PBS for 20 mins. Fixed cells were blocked with 1% bovine serum albumin for 1 hour at room temperature prior to incubation with rabbit anti-FAK IgG (Sigma, diluted 1 : 200 in PBS) overnight in the refrigerator. After incubation with primary antibody, cells were washed with PBS and incubated with Alexa Fluor 555 donkey anti-rabbit IgG (Invitrogen, diluted 1 : 200 in PBS) and Alexa 488 phalloidin (Invitrogen, diluted 1 : 200 in PBS) for 1 hour at room temperature. Samples were mounted using Cytoseal (Sigma) and imaged on an Olympus IX71 inverted microscope using a 40x objective. Raw images were processed using ImageJ software (NIH).

### 2.6. Image Acquisition

Live cell imaging was performed using Olympus IX71 microscope. Images were recorded with a CCD camera (QImaging) interfaced to image acquisition software (Image-Pro Express 6.3). For analysis of cell shape on the different ECM-coated substrates, at least 50 cells were analyzed for every condition and the experiment repeated twice. Deadhesion measurements were performed as described previously [30]. Briefly, media was aspirated, and cells were washed with PBS before being incubated with warm trypsin (0.25% trypsin with 0.02% EDTA, Sigma). Images were acquired every 5-6 sec at 20x magnification until cells became rounded but were still attached to the underlying substrate. To quantify deadhesion, cell-substrate contact area was determined by tracing the outline of the cell at different time points using ImageJ (NIH). Deadhesion dynamics were quantified by fitting the time-dependent normalized area ($\overset{-}{A}(t)=\left(A_{\text{initial}}-A(t)\right)/\left(A_{\text{initial}}-A_{\text{final}}\right)$) to a sigmoidal curve ($\overset{-}{A}=1-1/\left(1+e^{(t-\tau_{1})/\tau_{2}}\right)$) to obtain estimates of the two time constants *τ*_1_ and *τ*_2_, respectively. Statistical significant differences between time constants across different conditions were assessed using Student's *t*-test.

## 3. Results

### 3.1. Quantification of Surface Adsorption of Collagen and Fibronectin

**Figure 1:**
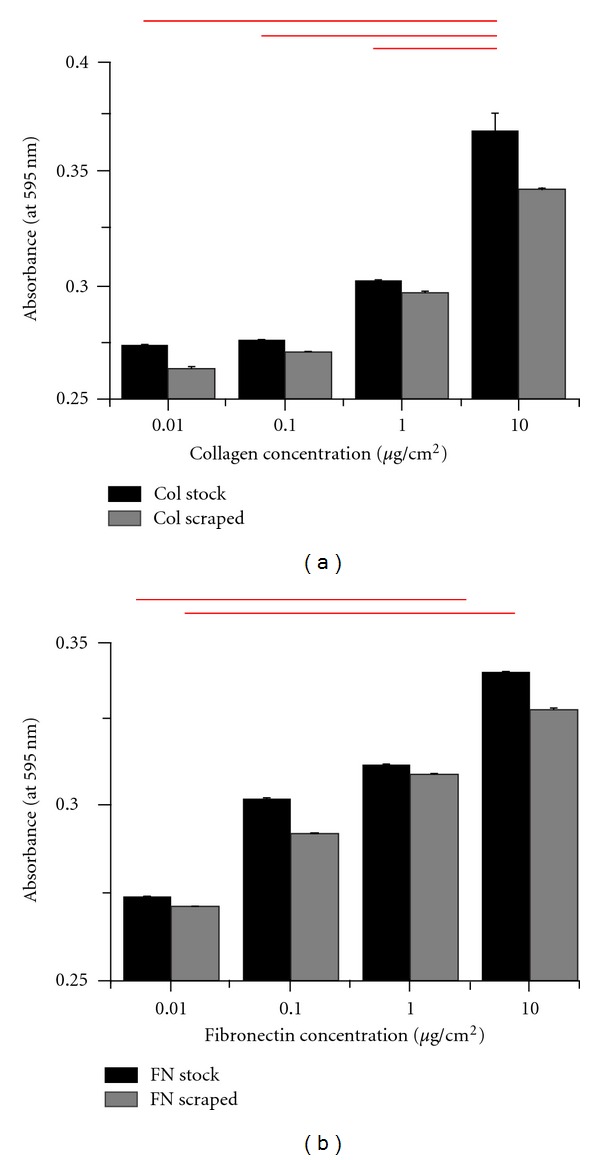
Quantification of ECM surface adsorption at different densities. Surface adsorption of collagen and fibronectin at different densities was quantified using UV spectrophotometer (Perkin Elmer). Surfaces were incubated with stock solutions of collagen and fibronectin at the theoretical densities of 0.01, 0.1, 1.0, and 10.0 *μ*g/cm^2^, respectively, overnight at 4 degrees. The surface-adsorbed ECM protein at different densities were extracted and quantified to determine the amount of attached protein. The absorbance values of the stock solutions (grey bars) were compared with those of the adsorbed solutions (black bars) at different densities. While higher absorbance values were obtained for higher ECM density, similar absorbance values between stock solutions and adsorbed solutions at different densities indicate >90% adsorption across all the different conditions. Bars indicate statistical significance (*P < 0.05).

To systematically study the influence of ECM ligand density on deadhesion dynamics of MDA-MB-231 cells, glass coverslips were coated with collagen or fibronectin at coating densities of 0.01, 0.1, 1.0, and 10 *μ*g/cm^2^, respectively. The adsorbed proteins were extracted by scraping and reconstituted in equal volume of buffer as that of the stock volume, and the degree of surface attachment of ECM proteins characterized using UV spectrophotometer. The extent of surface adsorption was assessed by comparing the absorbance of stock solutions with those of the adsorbed solutions. Quantitative measures of absorbance (at 595 nm) of the stock solutions showed an increase in absorbance with increase in densities, for both collagen and fibronectin (Figure 1). Further, absorbance readings of the adsorbed solutions were 95–98% of the stock solutions indicating a near 100% surface adsorption of the ECM proteins.

### 3.2. ECM Density-Dependent Spreading Responses on Collagen- and Fibronectin-Coated Substrates

**Figure 2:**
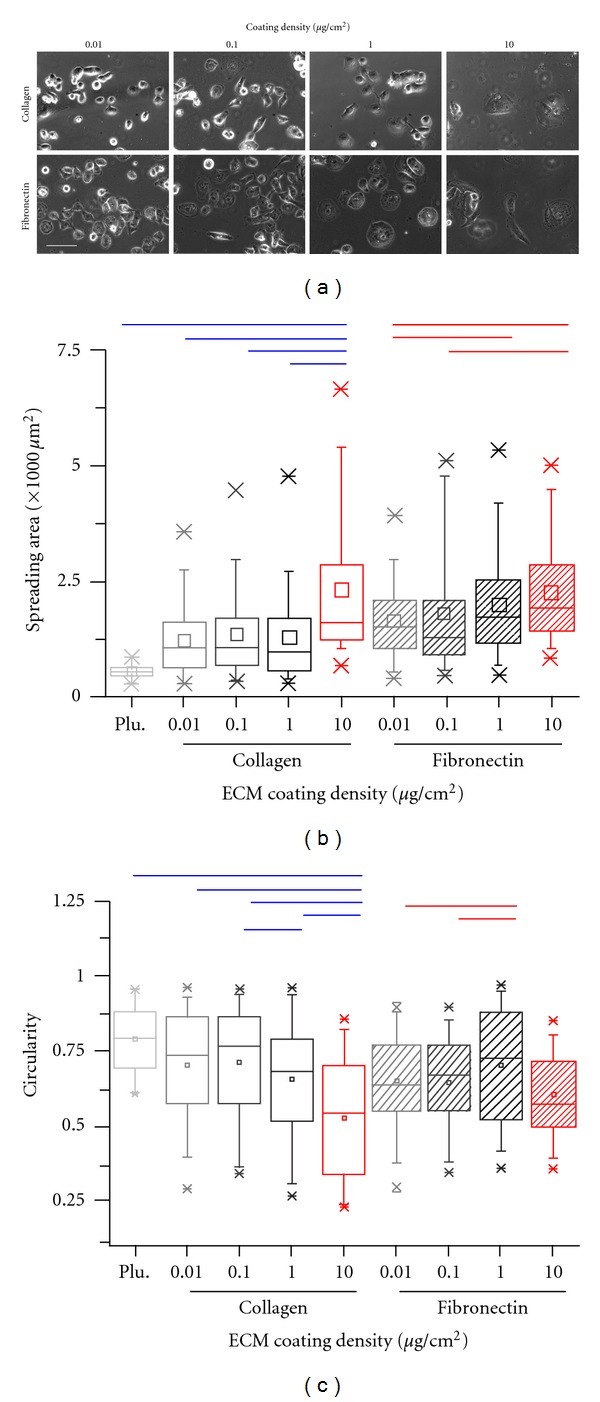
ECM density-dependent cell spreading of MDA-MB-231 cells on collagen- and fibronectin-coated surfaces. (a) Representative phase contrast images of MDA-MB-231 cells cultured on collagen and fibronectin-coated coverslips at different densities for 24 hours. Scale bar = 100 *μ*m. (b) Box whisker plots of projected cell area on coverslips coated with collagen or fibronectin at varying densities. Pluronic-blocked coverslips (Plu.) without any matrix coating served as negative controls. Independent of choice of ECM protein, spreading was relatively insensitive to ligand density up to a coating density of 1 *μ*g/cm^2^ and increased thereafter. Bars indicate statistical significance (*P < 0.05). (c) Box whisker plots of circularity measures on coverslips coated with collagen or fibronectin at varying densities. Increase in ECM density was associated with a reduction in circularity on collagen-coated surfaces. Circularity exhibited a biphasic behavior on fibronectin-coated surfaces. Bars indicate statistical significance (*P < 0.05).

The highly invasive and metastatic human MDA-MB-231 breast tumor cells were chosen for our studies. These cells are classified as triple-negative as they do not express estrogen receptor, progesterone receptor, or HER2 [35]. Also, they are known to express the intermediate filament protein vimentin and lack the tumor suppressor E-cadherin [36]. To study the influence of ECM density on cell spreading, equal number of MDA-MB-231 cells were cultured on collagen- and fibronectin-coated glass coverslips of varying ECM densities for a period of 24 hours. Pluronic-blocked glass coverslips without any ECM coating served as negative controls to correct for spreading driven by passive adsorption of serum proteins and/or secretion of ECM proteins by cells themselves. The 5-fold higher spreading of cells even on the lowest 0.01 *μ*g/cm^2^ ECM-coated surfaces compared to Pluronic-blocked surfaces suggested that there was minimal adsorption of serum proteins at the 24-hour time point (Figure 2(b)). Representative phase contrast images of cells on different ECM-coated surfaces suggested that cell spreading was sensitive to both ECM density and ECM composition (Figure 2(a)). In spite of the wide heterogeneity in cell responses to ECM density as observed from the box whisker plots, the increase in ECM density led to increase in cell spreading beyond a threshold density of 1 *μ*g/cm^2^, with the highest spreading observed on the 10 *μ*g/cm^2^ surfaces. While spreading area changes were similar on both collagen and fibronectin, measures of circularity on different ligand-coated surfaces revealed differences in the pattern of spreading (Figure 2(b)). While increase in collagen density was associated with reduced circularity, that is, increased cell elongation, cells plated on fibronectin exhibited a biphasic trend in cell shape with increasing the ligand concentration, with maximal cell elongation on 1 *μ*g/cm^2^ surfaces. Collectively, these results suggest that while changes in cell spreading area may not be drastic, the mode of spreading may vary depending on both ECM composition and density.

### 3.3. Effect of ECM Density on Cytoarchitecture and Focal Adhesions of MDA-MB-231 Cells

**Figure 3:**
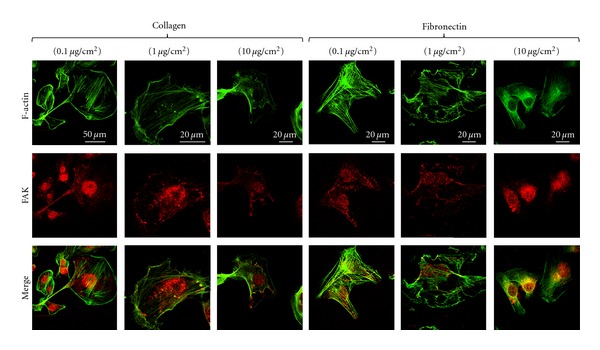
ECM density-dependent cytoskeletal architecture and focal adhesions of MDA-MB-231 cells. MDA-MB-231 cells were cultured on varying density collagen and fibronectin-coated surfaces. Cells were fixed after 24 hours in culture and stained for F-actin (green) and focal adhesion kinase (FAK) (red). Merged images show the colocalization of the actin fibers and FAK at the points of focal adhesion formation (yellow). Prominent stress fibers were observed across all the conditions. Cells plated on 0.1 *μ*g/cm^2^ collagen-coated substrates were more rounded and exhibited nuclear/perinuclear FAK localization. At higher collagen concentrations, cells became more elongated with FAK-positive peripheral focal adhesions. FAK localization on fibronectin-coated substrates exhibited an opposite trend to that on collagen-coated substrates with peripheral adhesions at lower densities and nuclear/perinuclear staining at higher densities.

To gain additional insight into the density-dependent differences in spreading, the steady- state assembly of focal adhesions and the actin cytoskeleton were assessed using immunofluorescence (Figure 3). For this, cells cultured on 0.1, 1.0, and 10.0 *μ*g/cm^2^ surfaces were stained for F-actin and FAK, respectively. While prominent stress fibers were observed across all conditions, ECM density-dependent differences in the organization of stress fibers was observed. For example, compared to the existence of both central and peripheral stress fibers on 0.1 and 1.0 *μ*g/cm^2^ collagen-coated surfaces, there was a prevalence of peripheral stress fibers on the 10.0 *μ*g/cm^2^ surfaces. Concomitant with these changes in the actin cytoskeleton, FAK exhibited a relocalization from the nuclear/perinuclear regions at low density to peripheral focal adhesions at high density. These changes are consistent with the increased cell spreading and elongation at the highest density. On fibronectin-coated surfaces, FAK localization exhibited an opposite trend with peripheral staining at lower densities to nuclear/perinuclear staining at higher densities. Further, thicker stress fibers on the 1.0 *μ*g/cm^2^ surfaces were replaced by a finer actin network on the 10.0 *μ*g/cm^2^ surfaces. Taken together, these results illustrate that while cell spreading area is not greatly sensitive to changes in ECM density, the distribution of focal adhesions and the actin network architecture is tuned closely by ECM density and composition.

### 3.4. ECM Density-Dependent Deadhesion Dynamics of MDA-MB-231 Cells

**Figure 4:**
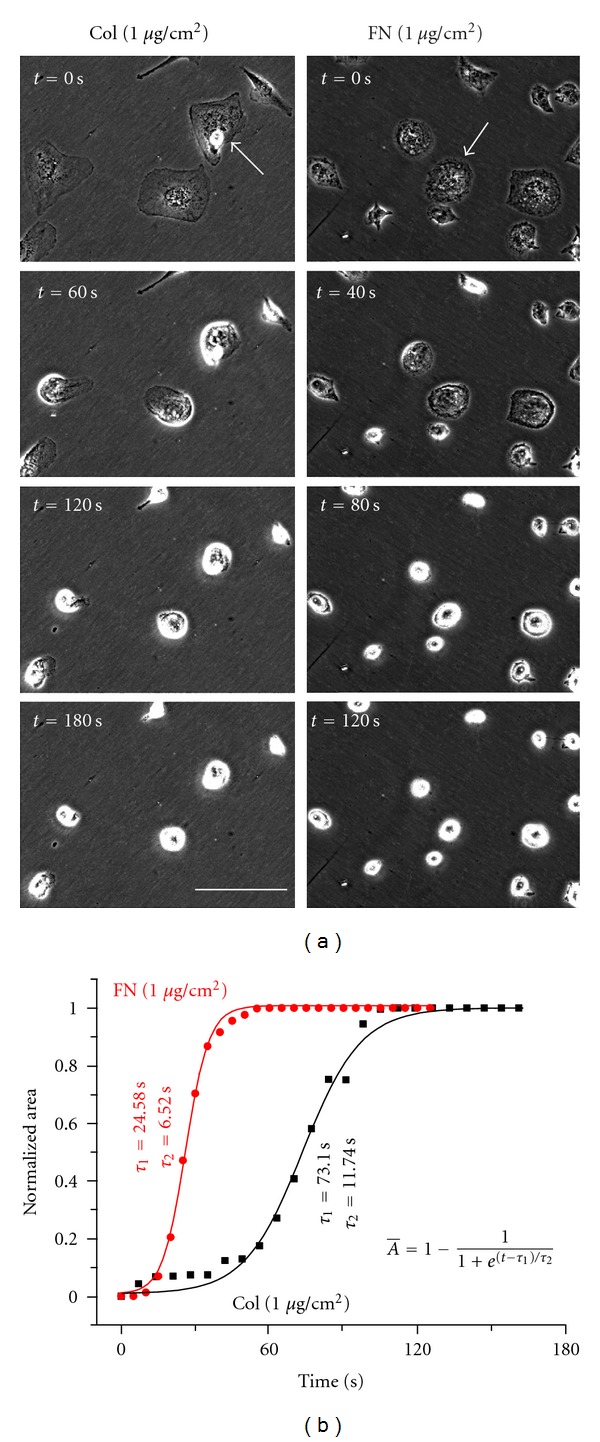
Deadhesion dynamics of MDA-MB-231 cells on collagen- and fibronectin-coated surfaces at a theoretical density of 1 *μ*g/cm^2^. (a) Media was removed and cells were washed with PBS prior to incubation with warm trypsin. Cells were imaged immediately upon addition of trypsin every 5 or 6 seconds at low magnification (20x) till cells rounded up but remained attached to the substrate. Scale bar = 100 *μ*m. (b) Deadhesion of the cells indicated by arrows was quantified by plotting normalized area as a function of time. The normalized data were fit to a Boltzmann equation to obtain the time constants *τ*_1_ and *τ*_2_, as indicated next to the fits (lines).

While drastic differences in cell spreading on different density surfaces were not observed, subtle changes in the distribution of focal adhesions and the actin network architecture suggest that differences may exist in the biophysical state of the cell across the different conditions. To directly determine the influence of ligand density on cell mechanics, the contractility of MDA-MB-231 cells was assessed using the trypsin deadhesion assay. Briefly, cells were washed with PBS and incubated with warm trypsin. Images were acquired every 5-6 secs till the cells rounded up but remained attached to the substrate. As seen in Figure 4(a), for the same coating density (1 *μ*g/cm^2^), cells on fibronectin-coated substrates were found to round up faster compared to cells on collagen-coated substrates. This was quantitatively confirmed by plotting the normalized cell area as a function of time and fitting the experimental deadhesion curves with the Boltzmann equation to obtain the time constants *τ*_1_ and *τ*_2_ (Figure 4(b)), whereas *τ*_1_ ~ 25 sec for the cell cultured on fibronectin and *τ*_1_ ~ 75 sec for the cell cultured on collagen, indicative of a threefold faster initial detachment response on fibronectin-coated surfaces. Similarly, compared to *τ*_2_ ~ 12 sec on collagen, *τ*_2_ ~ 6.5 sec on fibronectin corresponded to a nearly 50% faster response. The deadhesion responses of the two cells on two different substrates seem to suggest that under identical ligand density, cells are more contractile on fibronectin-coated substrates compared to collagen-coated substrates.

**Figure 5:**
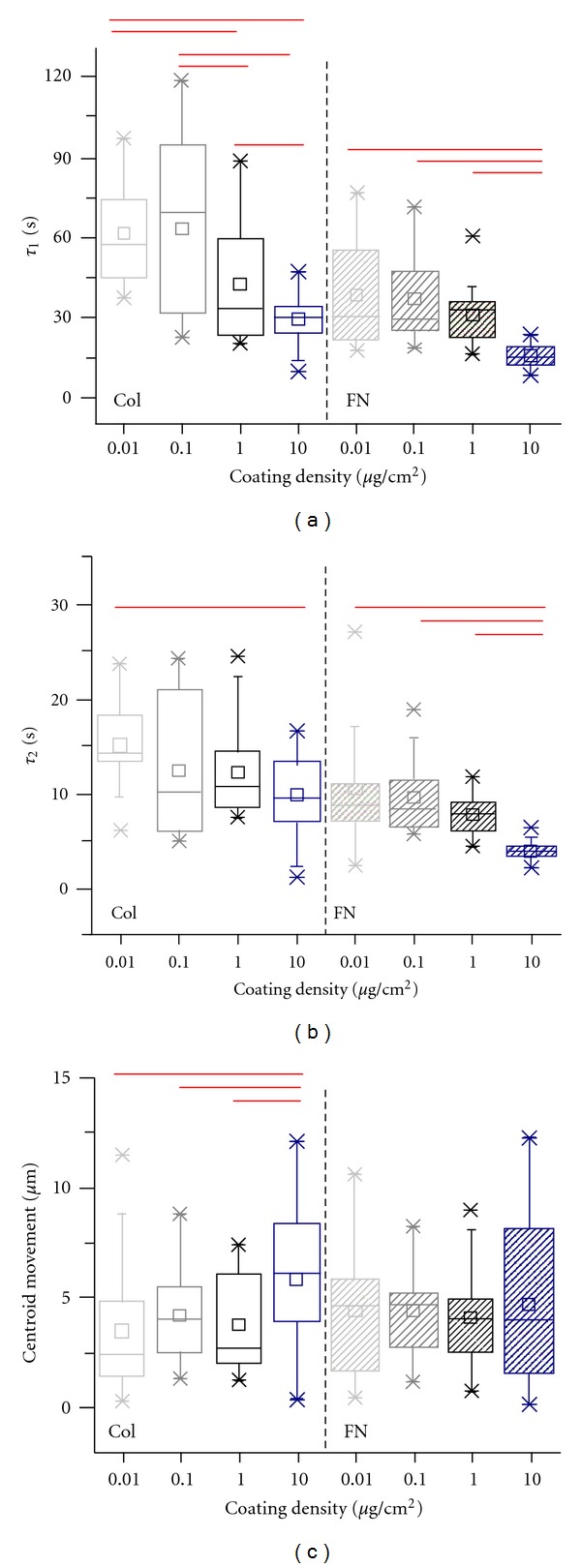
ECM density-dependent deadhesion dynamics of MDA-MB-231 cells on collagen and fibronectin-coated substrates. ((a), (b)) Box whisker plots of time constants of retraction (*τ*_1_ and *τ*_2_) on coverslips coated with collagen and fibronectin at varying densities. Faster deadhesion was observed at higher coating densities for both the ECM proteins. Bars indicate statistical significance (*P < 0.05). (c) The net movement of the cell centroid during the deadhesion process was quantified for individual conditions. Centroid movement remained constant for all the conditions except on the 10 *μ*g/cm^2^ collagen-coated surfaces where it was significantly higher (*P < 0.05).

To further probe if faster deadhesion on fibronectin-coated substrates holds true for all densities, we conducted deadhesion studies on collagen- and fibronectin-coated surfaces across all the different densities (Figures 5(a) and 5(b)). Interestingly, on collagen-coated substrates, MDA-MB-231 cells exhibited density-dependent deadhesion dynamics with *τ*_1_ ~ 60 sec on 0.01 and 0.1 *μ*g/cm^2^ surfaces, *τ*_1_ ~ 45 sec on 1.0 *μ*g/cm^2^ surfaces, and *τ*_1_ ~ 30 sec on 10 *μ*g/cm^2^ surfaces. While deadhesion was faster across all the densities on fibronectin-coated substrates compared to collagen-coated substrates, similar density dependence was also observed on fibronectin-coated substrates with *τ*_1_ ~ 35 sec on 0.01 and 0.1 *μ*g/cm^2^ surfaces, *τ*_1_ ~ 30 sec on 1.0 *μ*g/cm^2^ surfaces, and *τ*_1_ ~ 20 sec on 10 *μ*g/cm^2^ surfaces. Though density-dependent changes in *τ*_2_ were less dramatic, trends were similar to those of *τ*_1_ with lowest values obtained at highest densities.

In addition to tracking changes in cell area during deadhesion, we determined one more variable—net centroidal movement—from the deadhesion experiments (Figure 5(c)). This represents the net movement of the centroid of the cell during the process of deadhesion and is indicative of the extent of cell polarization. A nonzero centroidal movement of ~4 *μ*m was observed for all the conditions, except on 10 *μ*g/cm^2^ collagen-coated surfaces on which cells were most polarized as evident from the ~6.5 *μ*m centroidal movement.

### 3.5. Effect of Myosin II Inhibition on Deadhesion Dynamics of MDA-MB-231 Cells

**Figure 6:**
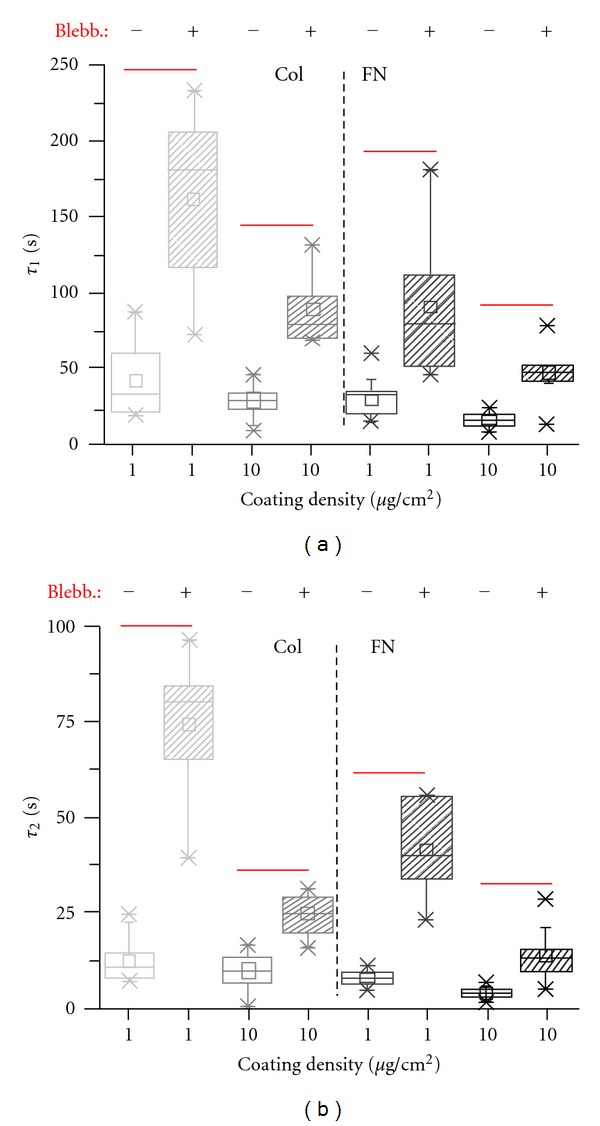
Effect of nonmuscle myosin II inhibition on deadhesion dynamics of MDA-MB-231 cells. MDA-MB-231 cells were incubated with 5 *μ*M blebbistatin for 30 mins prior to deadhesion. ((a), (b)) Blebbistatin treatment led to a significant delay in deadhesion dynamics of MDA-MB-231 cells across all the conditions. Differences in *τ*_1_ and *τ*_2_ between untreated cells and blebbistatin-treated cells were statistically significant for all the conditions (*P < 0.05).

Previous studies of deadhesion dynamics have demonstrated the prominent role of contractility in driving deadhesion [30]. To test if myosin-based contractility is the major determinant of the deadhesion response of MDA-MB-231 cells, deadhesion experiments were performed on cells that were incubated with the nonmuscle myosin II inhibitor blebbistatin for 30 minutes at a dosage of 5 *μ*M. The drug studies were restricted to 1 and 10 *μ*g/cm^2^ ligand-coated surfaces, where faster deadhesion was observed. Blebbistatin treatment significantly delayed the deadhesion response of MDA-MB-231 cells both on collagen-coated and on fibronectin-coated surfaces at both ligand densities (Figure 6). Specifically, a 3-4-fold increase in *τ*_1_ (Figure 6(a)) and a 2–5-fold increase in *τ*_2_ (Figure 6(b)) were observed across the different conditions. Collectively, these results demonstrate the role of actomyosin contractility in setting deadhesion time scales.

### 3.6. Spreading Response of MDA-MB-231 Cells on Mixed ECM Surfaces

**Figure 7:**
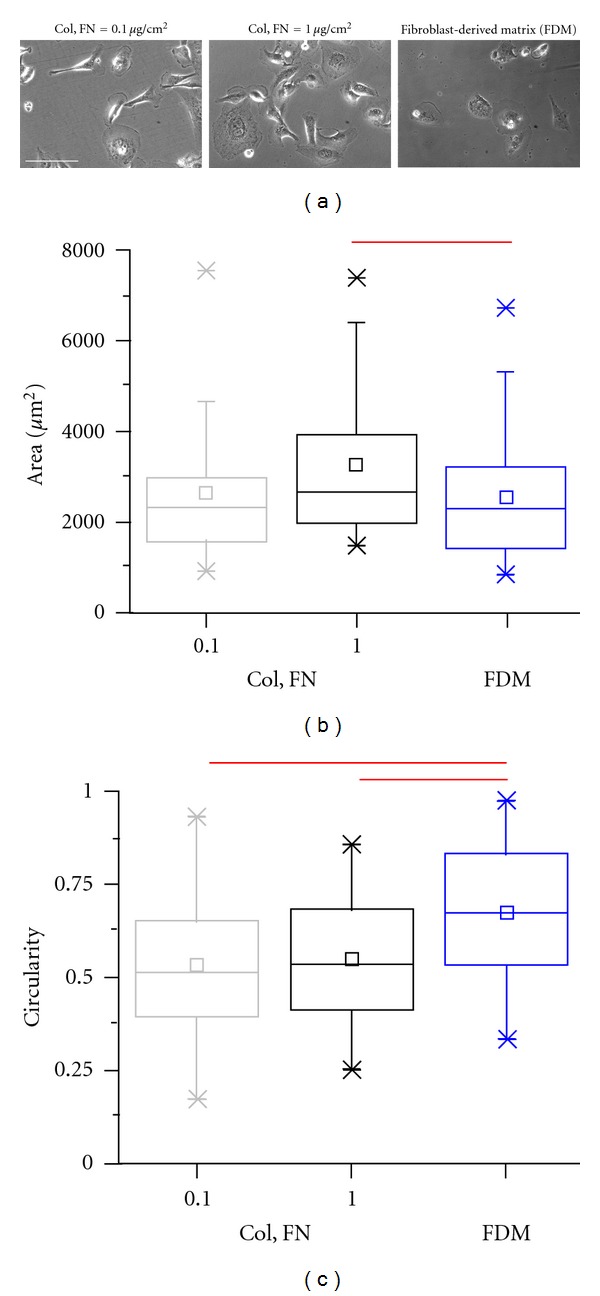
Morphometric analysis of MDA-MB-231 cells on mixed ECM surfaces. (a) Representative phase contrast images of MDA-MB-231 cells cultured for 24 hours on coverslips coated with 0.1 *μ*g/cm^2^ collagen and fibronectin (Col, FN = 0.1 *μ*g/cm^2^), 1.0 *μ*g/cm^2^ collagen and fibronectin (Col, FN = 1.0 *μ*g/cm^2^), and on fibroblast-derived matrix (FDM). Scale bar = 100 *μ*m. (b) Box whisker plots of projected cell area on different mixed ECM surfaces. Cells spread to a lesser extent on FDMs. Maximal spreading was observed for Col, FN = 1.0 *μ*g/cm^2^ (*P < 0.05). (c) Box whisker plots of circularity measures on different mixed ECM surfaces. Cells were statistically more rounded on FDMs compared to the other conditions (*P < 0.05).

While the above studies have provided us with important insights into the spreading and deadhesion response of breast cancer cells on collagen-coated and fibronectin-coated substrates at varying densities, in reality, *in vivo* matrices are composed of more than one ECM protein. Further, in addition to matrix composition, *in vivo* matrices provide various spatial cues to cells in the form of matrix topography and dimensionality [34, 37]. Taking cognisance of these facts, we repeated our spreading studies in matrices composed of more than one ECM protein. Two of these were obtained by combining both collagen and fibronectin at defined densities of 0.1 (Col, FN = 0.1 *μ*g/cm^2^) and 1.0 *μ*g/cm^2^ (Col, FN = 0.1 *μ*g/cm^2^), respectively. The third matrix, fibroblast-derived matrix (FDM), was a matrix directly secreted by NIH 3T3 fibroblasts and closely mimics the composition and organization of *in vivo* 3D matrices [33, 38]. Representative phase contrast images of cells were indicative of differences in the spreading responses on the three matrices (Figure 7(a)). Maximum spreading was observed on the 1.0 *μ*g/cm^2^ dual ECM surfaces. Similar spreading was observed on the 0.1 *μ*g/cm^2^ dual ECM surfaces and the FDMs (Figure 7(b)). However, cells on the FDMs were significantly more rounded compared to the two dual ECM surfaces, suggestive of altered cytoskeletal organization.

### 3.7. Deadhesion Dynamics of MDA-MB-231 Cells on Mixed ECM Surfaces

**Figure 8:**
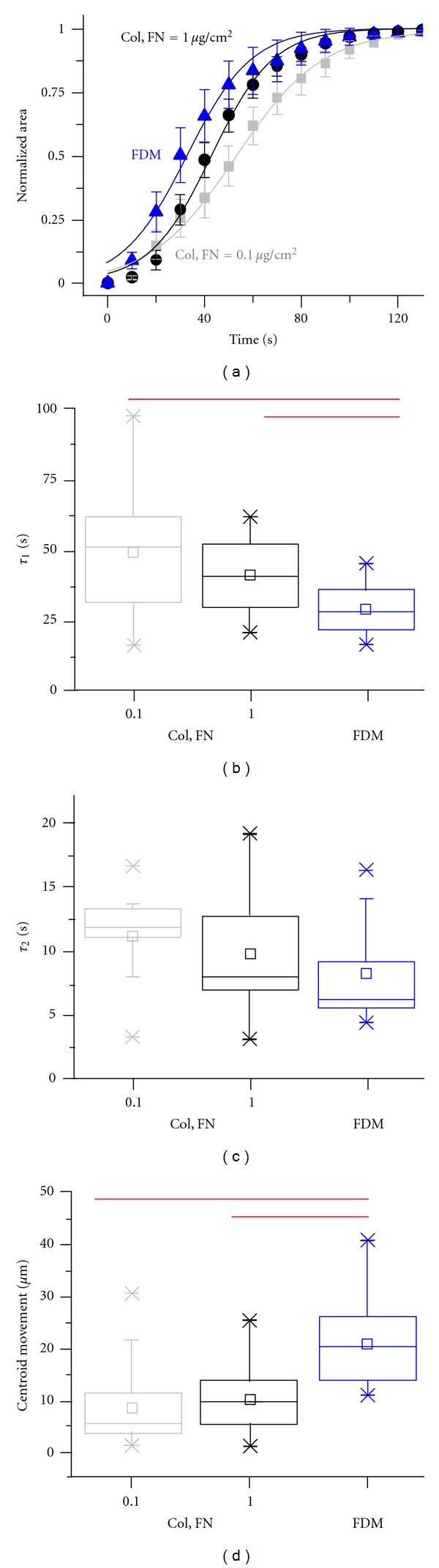
Deadhesion dynamics of MDA-MB-231 cells on mixed ECM surfaces. (a) Quantification of cell shape changes during deadhesion on ECM surfaces of varying composition. Plots of normalized cell area for cells cultured on coverslips coated with Col, FN = 0.1 *μ*g/cm^2^, Col, FN = 1.0 *μ*g/cm^2^, and on FDMs. ((b), (c)) Box whisker plots of retraction time constants of cells plated on mixed ECM surfaces. Increase in coating density led to faster deadhesion, with the fastest deadhesion observed on FDMs (*P < 0.05). (d) Box whisker plots of centroid movement of cells during deadhesion on mixed ECM surfaces. While centroid movement remained unchanged on the two different Col/FN surfaces, significantly higher centroid movement was observed on FDMs.

To further probe differences in the cellular mechanics on the mixed ECM surfaces, deadhesion experiments were repeated on these three matrices. The detachment response on all the three matrices was sigmoidal similar to earlier studies highlighting the generality of the deadhesion process. On the dual ECM surfaces, faster deadhesion was observed on the 1.0 *μ*g/cm^2^ higher-density surfaces (Figure 8(a)), similar to the density-dependent deadhesion responses observed on individually collagen-coated and fibronectin-coated surfaces (Figures 5(a) and 5(b)). Interestingly, fastest deadhesion was observed on the FDMs. Compared to *τ*_1_ ~ 50 sec on the 0.1 *μ*g/cm^2^ dual ECM surfaces, *τ*_1_ ~ 25 sec on the FDMs represents a statistically significant difference of 50% (Figure 8(b)). Similarly, *τ*_2_ decreased by ~40% from ~12.5 sec on the 0.1 *μ*g/cm^2^ dual ECM surfaces compared to ~7.5 sec on the FDMs (Figure 8(c)). The deadhesion time constants on the 1.0 *μ*g/cm^2^ dual ECM surfaces were midway between the values observed on the 0.1 *μ*g/cm^2^ dual ECM surfaces and those observed on the FDMs. While deadhesion time scales were different for the two dual ECM surfaces, the centroidal movement remained unchanged indicative of similar extents of cell polarization on these two matrices (Figure 8(d)). Most strikingly, the centroidal movement of ~20 *μ*m on FDMs was nearly 2-fold of the centroidal movement observed on the dual ECM surfaces. In addition to illustrating synergistic modulation of cell contractility by mixed ECM ligands, the results on FDMs highlight the role of ECM dimensionality in regulating cellular contractility and polarization, factors that directly contribute to cancer invasiveness.

## 4. Discussion

In this study, we sought to determine the combined influence of ECM composition and ECM density on the mechanics of breast cancer cells. We have shown that increase in ECM density leads to increased cell spreading beyond a threshold density that corresponds to 1.0 *μ*g/cm^2^ in our studies. While the density-dependent spreading responses are similar on collagen and fibronectin-coated surfaces, cell shape changes are dependent on ECM composition. Probing cell contractility using trypsin induced deadhesion, we have shown that increase in ECM density activates cell contractility. On hybrid matrices containing both collagen and fibronectin, cells spread more than those on either collagen-coated or fibronectin-coated matrices but exhibit an average deadhesion response. Lastly, we have shown that cells on FDMs possess higher contractility and polarization compared to mixed ECM surfaces, highlighting the direct influence of ECM organization on cell mechanics. Together, our results demonstrate that ECM composition, density, and 3D organization collectively regulate cell contractility.

Traction force microscopy is a widely used technique for measuring the contractility of adherent cells [39, 40]. Using this method in conjunction with magnetic twisting cytometry, Wang and coworkers demonstrated the linear relationship between cell stress and cell stiffness, with increase in stress associated with cell stiffening [27]. This method has been successfully used by multiple groups to measure traction forces in a range of different cell types ranging from fibroblasts to cancer cells to stem cells [22, 41–45]. However, measurement of traction forces requires embedding of fluorescent beads in the substrate. While this is easily achievable with engineered matrices like polyacrylamide hydrogels, this requirement becomes a handicap while trying to measure traction forces in more *in vivo* mimetic contexts, like fibroblast-derived matrices [33]. Also, traction forces cannot be measured on rigid surfaces like glass coverslips, where a lot of cell biology work gets carried out. Since the deadhesion assay does not require any preprocessing of the substrate, therefore, it can be used both on engineered matrices and *in vivo* mimetic matrices. Given the simplicity and robustness of this technique, we anticipate wider usage of this technique by the cell mechanics community.

Cell retraction following trypsin-induced deadhesion is dictated both by the magnitude of contractile stress in the cell as well as the strength of adhesion to the substrate. While higher contractility is expected to cause faster deadhesion for constant adhesion strength, stronger adhesion is expected to lead to delayed deadhesion for identical levels of stress. Since adhesion and contractility are closely related in adherent cells [46, 47], deadhesion response is expected to be dictated by the degree of crosstalk between adhesion and contractility. Previous studies conducted with U373 MG human glioblastoma cells on fibronectin-coated surfaces of increasing density revealed that deadhesion was more sensitive to changes in contractility compared to ligand density [31]. In this paper, we have obtained similar results with MDA-MB-231 breast cancer cells both on collagen-coated and fibronectin-coated surfaces, with faster deadhesion observed on higher-density surfaces. 

Comparison of spreading and deadhesion dynamics of breast cancer cells on collagen-coated and fibronectin-coated surfaces offers interesting insights. On collagen-coated and fibronectin-coated surfaces, spreading remained unchanged up to a coating density of 1 *μ*g/cm^2^ and increased significantly on the 10 *μ*g/cm^2^ coated surfaces. These results are consistent with previous reports that show a weak dependence of cell spreading on ECM density when cultured on stiff substrates [13]. However, differences exist between the density-dependent cell shapes on these two surfaces as evidenced in our circularity measures. Interestingly, we observe that cell deadhesion measurements are more sensitive to changes in ECM density compared to cell spreading. Further, faster deadhesion was observed on fibronectin-coated surfaces compared to collagen-coated surfaces, suggesting that increased contractility is associated with increased spreading, as has been observed in fibroblasts and endothelial cells [11, 48]. Faster deadhesion beyond a critical coating density (0.1 *μ*g/cm^2^ in our case) is indicative of a threshold density required for integrin clustering, formation of focal adhesions, and buildup of traction stresses.

Our experimental data suggests that the pattern of deadhesion can also provide us with useful information pertaining to the extent of cell polarization. Cell migration is a highly regulated process that consists of front-to-back cell polarization, actin-driven protrusion of the leading edge, adhesion formation, generation of traction stresses, and rear-edge retraction [49, 50]. Several studies of cell migration in various different cell types have shown that ECM density or stiffness modulates cell speed in a biphasic manner [11, 13, 51–53]. Owing to cell polarity, motile cells have stronger adhesions at the leading edge and weaker at the trailing edge. In addition, larger traction forces are exerted at the leading edge compared to the trailing edge. Such asymmetry in adhesion strength and/or contractility is manifested as a nonzero centroidal movement during the deadhesion process, with the magnitude of centroidal movement indicative of the extent of polarization. Our results indicate that polarization is uniform across all the conditions except on 10 *μ*g/cm^2^ collagen-coated surfaces on which cells are maximally polarized.

Our experiments with the hybrid ECM surfaces provide additional insight into the effects of multiple integrin subtypes binding to multivalent ECM signals on cell mechanics. Earlier studies using engineered mixed ligand surfaces have demonstrated that the presentation of multiple integrin-binding ligands cooperatively enhances cell adhesion strength, proliferation, and adhesive signaling [54]. In agreement with this study, cell spreading on the mixed ECM surfaces was found to be significantly enhanced compared to those on single ligand surfaces, for the same coating density. However, comparison of the deadhesion time constants on mixed matrices with those on single ligand surfaces indicates that on the mixed matrices cells engage collagen and fibronectin in equal measure. For example, *τ*_1_ ~ 50 sec on the 0.1 *μ*g/cm^2^ dual ECM surfaces represents the average of *τ*_1_ ~ 60 sec on the 0.1 *μ*g/cm^2^ collagen-coated surfaces and *τ*_1_ ~ 40 sec on the 0.1 *μ*g/cm^2^ fibronectin-coated surfaces, indicative of an average response on the mixed ECM surfaces. Together, these results demonstrate the importance of considering the interplay of multiple integrins on cell mechanics and downstream signaling.

To better understand cancer progression, significant effort is focused on fabricating *in vitro* systems that recapitulate many of the physical and chemical features of the tumor microenvironment. FDMs represent one of the recently developed natural matrix systems that closely mimic *in vivo* matrices [33, 34, 38, 55]. These matrices have been shown to be more than 6-fold effective in mediating cell adhesion compared to 2D substrates coated with collagen and fibronectin and 3D collagen gels [34]. Moreover, faster migration on these matrices compared to migration on 2D surfaces has been attributed to the fibrillar organization of these matrices that provide a directional cue to the cells [37]. Consistent with these findings, our deadhesion results highlight the high degree of polarization of cells on FDMs, compared to mixed ECM surfaces, and the hypercontractile phenotype of these cells on the FDMs.

In summary, our results highlight the synergistic modulation of contractility of breast cancer cells by mixed ECMs composed of collagen and fibronectin. Our results of cell contractility on FDMs demonstrate that contractile mechanics is tuned simultaneously by the physical and chemical features encoded by the ECM and highlight the necessity of studying cancer cells properties in more physiologically relevant matrix systems. Future work will involve taking a closer look at the relationship between cell shape and deadhesion kinetics.
